# Underlying mechanisms and drug intervention strategies for the tumour microenvironment

**DOI:** 10.1186/s13046-021-01893-y

**Published:** 2021-03-15

**Authors:** Haoze Li, Lihong Zhou, Jing Zhou, Qi Li, Qing Ji

**Affiliations:** 1 Department of Medical Oncology and Cancer Institute, Shuguang Hospital, Shanghai University of Traditional Chinese Medicine, Shanghai, 201203 China; 2 Academy of Integrative Medicine, Shanghai University of Traditional Chinese Medicine, Shanghai, 201203 China

**Keywords:** TME, Cancer development, Molecular targets, Drug intervention strategies

## Abstract

Cancer occurs in a complex tissue environment, and its progression depends largely on the tumour microenvironment (TME). The TME has a highly complex and comprehensive system accompanied by dynamic changes and special biological characteristics, such as hypoxia, nutrient deficiency, inflammation, immunosuppression and cytokine production. In addition, a large number of cancer-associated biomolecules and signalling pathways are involved in the above bioprocesses. This paper reviews our understanding of the TME and describes its biological and molecular characterization in different stages of cancer development. Furthermore, we discuss in detail the intervention strategies for the critical points of the TME, including chemotherapy, targeted therapy, immunotherapy, natural products from traditional Chinese medicine, combined drug therapy, etc., providing a scientific basis for cancer therapy from the perspective of key molecular targets in the TME.

## Background

Cancer has been a difficult problem worldwide. In the United States, the overall 5-year relative survival rate of all cancers diagnosed between 2009 and 2015 is 67%, and the United States is expected to have 1,806,590 new cancer cases and 606,520 cancer deaths in 2020 [1]. Therefore, to effectively treat cancer and prevent cancer recurrence and metastasis, researchers have studied its treatment for a long time. Presently, the main cancer treatment strategies include surgery, chemotherapy, radiotherapy, targeting therapy, immunotherapy, traditional Chinese medicine, etc., but their effects are not satisfactory. For example, the five-year recurrence-free survival rate of adenocarcinoma is 30% [2]. In addition, although the clinical efficacy of gemcitabine^+^NAB^−^ paclitaxel and other multi-drug regimens in the treatment of pancreatic cancer has been improved, it has not reached a satisfactory level [3]. Based on this, researchers not only pay attention to the treatment of cancer itself but also gradually try to change the TME through different strategies to indirectly achieve cancer treatment.

Currently, cancer treatment strategies targeting the TME are mainly focused on different molecular targets, special cells or intercellular ingredients in the TME through the related effects of chemotherapy, targeted therapy, immunotherapy, traditional Chinese medicine and even nanotechnology on TME to inhibit the promoting effect of the TME on cancer occurrence, development and metastasis. Cancer not only occurs in a complex tissue environment but also depends on the continuous spread, invasion and migration of the tissue environment.

The TME originates from the idea of “seed and soil” proposed by Stephen Paget, which holds that metastasis depends on the interaction between the “seed” (cancer cell) and “soil” (host microenvironment) [4]. Similarly, evidence has shown that primary cancers can induce secondary organs to gradually form a supportive microenvironment called the pre-metastatic niche [5]. Therefore, the TME is one of the key factors that affect cancer metastasis and growth.

As the importance of the TME to cancer development and metastasis is gradually known, the prevention and treatment of cancer through targeting the TME has become a research hotspot. In this paper, we reviewed our understanding of the TME, described its biological and molecular characteristics at different stages of cancer development, and discussed in detail the intervention strategies of key points of the TME, hoping to provide a scientific basis for cancer treatment from the perspective of the TME.

## Critical links and mechanisms of the TME

In 1993, Anderson and Whitesid formally proposed the concept of the “TME” [6], indicating that the TME is the internal environment for the generation and life of cancer cells, which is mainly constituted by cancer cells, locally infiltrated immune cells, mesenchymal cells and their secreted active mediators, providing “fertile soil” for the proliferation, development, metastasis and other malignant biological behaviours of cancer cells. The TME is a complex system [7] that consists of the biological characteristics of hypoxia and low pH, blood vessels, high permeability, inflammatory response and immunosuppression. Among them, cancer cells communicate with the microenvironment and interact with each other, resulting in a high degree of cell proliferation and metastasis [8]. Therefore, the TME is considered to be an important cause of cancer proliferation, invasion, migration, adhesion and neovascularization.

### Primary TME

**Fig. 1:**
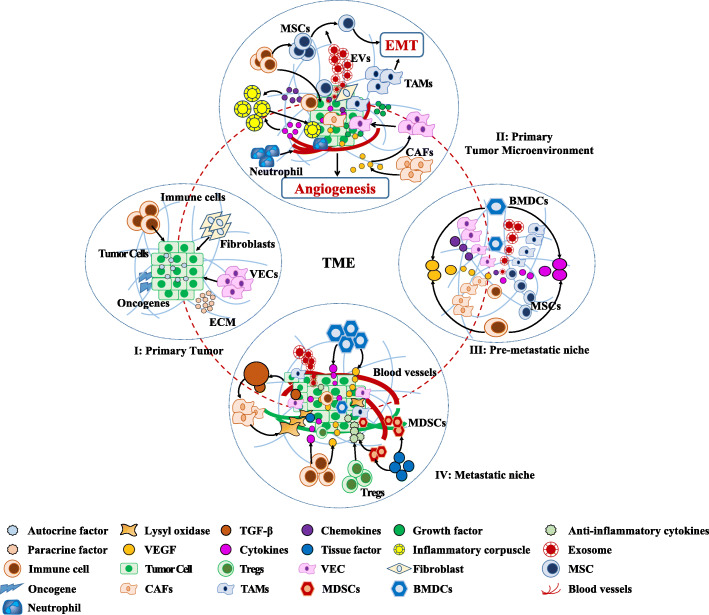
Multiple stages of the TME in cancer progression. (I) TME in the budding stage of primary cancer: Oncogene activation leads to the conversion of normal cells to cancer cells, accompanied by the initial microenvironment formation in primary cancer sites containing fibroblasts, immune cells, vascular endothelial cells (VECs), etc. (II) TME in the progressing stage of primary cancer: inflammatory cells (producing chemokines and cytokines), neutrophils, tumour-associated macrophages (TAMs, producing carcinogenic proteases, cytokines and growth factors, and angiogenic factors), VECs, cancer-associated fibroblasts (CAFs, producing vascular endothelial growth factor, VEGF), extracellular matrix (ECM), etc. (III) Pre-metastatic niche: macrophages, platelets, mesenchymal stem cells, bone marrow-derived dendritic cells (BMDCs), immune cells (producing inflammatory cytokines, growth factors and angiogenic factors), etc. (IV) Metastatic niche: myeloid-derived suppressor cells (MDSCs, producing tissue factors and anti-inflammatory cytokines), Treg cells (producing anti-inflammatory cytokines), CAFs (producing transforming growth factor-1), etc.

Generally, the TME is mainly composed of immune cells, vascular cells, fibroblasts, etc. Recruiting the above cells to the primary cancer site would construct a special TME and provide soluble paracrine signals to promote cancer progression (Fig. 1).

#### Transcription factors

High levels of inflammatory mediators are mainly caused by oncogene mutations and transcription factor activation. For example, mutation of the cancer suppressor gene p53 promotes the occurrence of cancer [9]. Mice expressing mutant p53 have more invasive and metastatic cancers than mice without p53 and are extremely prone to many types of cancers, including lung adenocarcinoma, squamous cell carcinoma, hepatocellular carcinoma, renal transitional cell carcinoma and colorectal cancer. Many of these cancers are invasive or show evidence of distant metastasis [10]. In addition, many types of p53 mutations in cancer cells produce resistance to anticancer drugs [11]. During tumorigenesis and progression, the important paracrine and autocrine factors are mainly cytokines and chemokines, which recruit and activate a variety of inflammatory cells in the TME [12]. The transcription factor STAT3 participates in the unique immunosuppressive pancreatic TME and pancreatic cancer progression in many ways through its activity in a variety of cell types, such as cancer cells and immune cells [13]. For example, in pancreatic cancer, the activation of STAT3 promotes the transformation of monocytes into monocyte-derived myeloid suppressor cells (MDSCs), thus participating in the immunosuppression of the pancreatic TME, affecting cancer stem cells and promoting the interstitial characteristics of cancer cells [14]. In ovarian cancer, activation of STAT3 can induce macrophages to differentiate into the M2 phenotype [15]. STAT3 activity can inhibit the chemotaxis and activation of CD8^+^ T cells in melanoma and mediate the differentiation of inhibitory T regulatory (Treg) cells, thus promoting cancer progression [16]. In addition, in solid cancers such as lung cancer, pancreatic cancer and intrahepatic cholangiocarcinoma, cancer-associated fibroblasts (CAFs) use STAT3 activity to secrete cytokines. These cytokines can recruit additional immune cells and promote STAT3 activity in other types of cells in the TME [17, 18].

#### Stromal cells

In the TME, stromal cells play crucial roles in initiating and maintaining chronic angiogenesis. Cancer endothelial cells can release specific growth factors, called vascular secretory factors [19]. Endothelial cells can change existing blood vessels or form new vascular networks to regulate the supply of nutrients and oxygen to cancer, and participate in communication between surrounding areas and cancer cells through paracrine and paracrine signals [20, 21].

Tumour-associated macrophages (TAMs) secrete pro-angiogenic factors to activate endothelial cells [22]. At the same time, neutrophils enter the TME and play various biological functions, including promoting cancer angiogenesis and cancer cell proliferation [23, 24]. On the other hand, cancer may induce fibroblasts and macrophages in the TME to acquire tumourigenic function. For instance, TAMs support a variety of primary cancer phenotypes by releasing a large number of cytokines, growth factors and carcinogenic proteases to participate in paracrine signalling circulation. In colorectal cancer, TAMs activate the IL-6/STAT3/miR-204-5p pathway by secreting IL-6, which supports cancer progression, increases the resistance of colorectal cancer to chemotherapeutic drugs and reduces drug-induced apoptosis [25]. Cancer-associated fibroblasts CAFs can activate cancer-derived factors, such as fibroblast growth factor (FGF), platelet-derived growth factor (PDGF) and transforming growth factor (TGF), and excrete basement membrane components and extracellular matrix proteins. Moreover, CAFs can also secrete vascular endothelial growth factor (VEGF) to support angiogenesis [26]. Cancer inflammation-related fibroblasts in bladder cancer produce VEGF, including VEGFA and VEGFB, which bind to VEGF receptors (FLT1, KDR, MET and FLT4) on endothelial cells, promote angiogenesis, affect the proliferation of cancer cells and stromal cells, and may recruit immune cells into the cancer stage [27].

#### Extracellular matrix

The cancer-associated extracellular matrix (ECM) has obvious differences in the composition and number of different constituents (i.e., proteoglycans, glycoproteins, proteins, and polysaccharides) in contrast to the ECM in normal tissues. Some studies have shown that the reciprocity between cancer extracellular matrix and normal breast tissue cells results in information interchange and sustained overexpression of cancer-specific genes [28]. Likewise, extracellular matrix components, including hyaluronic acid, collagen, fibronectin and laminin, cause ECM refactoring in the primary site of breast cancer through the interaction between cancer cells and extracellular matrix components [29]. Chemokines suppress cancer immunity by regulating the recruitment of Treg cells into the TME, thus supporting cancer initiation, progression and metastasis [30].

Studies have shown that the activation of hepatic stellate cells (HSCs) can secrete angiopoietin-1 (ANG-1), thereby promoting angiogenesis in hepatocellular carcinoma [31]. The functions of some components in the extracellular matrix may be similar, such as targeting cancer blood vessels or cancer cells; therefore, it is of clinical significance to counter the components in the extracellular matrix.

#### Extracellular vesicles

Extracellular vesicles (EVs), including exosomes, can regulate many aspects of cancer biology. Many cancer or immune cell-derived EVs are involved in the entire process of cancer progression, such as angiogenesis, metastasis, immunity and resistance to anticancer treatments [32–34]. More specifically, Cianciaruso et al. detected that TAMs produce many EVs and affect the biological behaviour of other types of cells in the TME, which may be achieved through EVs fusion or membrane contact and functional molecular metastasis [35]. Our group confirmed that the primary cancer releases ITGBL1 (integrin β1)-enriched EVs and promotes the growth of distal metastatic cancers through the formation of a fibroblast niche. Specifically, in a colorectal cancer (CRC) model, the primary cancer releases ITGBL1-enriched EVs to activate fibroblasts in distant organs, promote the secretion of the pro-inflammatory cytokines to induce the constitution of pre-metastatic niche or facilitate the progression of metastatic neoplasms [36].

#### Others

Interestingly, the consumption of L-arginine in the TME controls the T cell immune response, and the T cell immune response is the basic mechanism of cancer cell immune escape [37]. Extracellular characteristics also contribute to cancer progression, such as high tissue hydraulic pressure, low partial pressure of oxygen, or changes in specific components of the extracellular matrix. In addition, a hypoxic TME is one of the common features of solid cancers. Exosomes mediate extensive bi-directional signal transduction among various cell types (cancer cells-cancer cells, cancer cells-stromal cells and stromal cells-stromal cells) in the hypoxic TME. They are considered to regulate hypoxia adaptation and reconstruct the microenvironment in return [38]. In lung cancer, plasma exosomes secreted by hypoxemic BMSCs can promote the invasion of lung cancer cells by activating the STAT3 signalling and EMT [39]. The TME contains leaky and constricted blood vessels, characterized by hypoxia and acidosis, allowing cancer cells to promote angiogenesis, connective tissue proliferation, and inflammation without control, leading to a vicious cycle that promotes disease progression [40]. The anoxic microenvironment is beneficial to glycolysis and lactic acid production of key enzymes of glycolysis and lactate dehydrogenase A (LDH-A). Excessive production of lactic acid leads to an acidic pH promoting cancer metastasis [41].

### Pre-metastatic niche

Before cancer cell metastasis, primary focal cancer cells can secrete a variety of cytokines in remote organs, affecting and changing the formation of the organ metastasis microenvironment (pre-metastatic niche) (Fig. 1). It is worth noting that all events that adjust the formation of the niche before metastasis are deemed to occur before the cancer cells reach the niche. Cancer cells prepare and form pre-metastatic niches in distal organs, which refer to the arrangement of pre-metastatic signals, including exosomes, growth factors, cytokines, etc., to regulate their position before cancer metastasis.

#### Exosomes

Exosomes are a subclass of extracellular vesicles that participate in cell-cell communication. The exosomes secreted by stromal and cancer cells can not only regulate cancer progression in the primary TME but also help the formation of a pre-metastatic inflammatory niche [42]. Exosomes containing various proteins, miRNAs and mRNAs can promote the formation of a pre-metastatic niche either by mediating the relationship between surrounding components and cancer cells or by diverting their contents to recipient cells [43]. Wortzel et al. found that these exosomes remodel the TME and boost cancer growth by delivering active molecules and RNA to other cells [44]. Exosomal miR-21 promotes liver metastasis by activating macrophages to form pro-inflammatory phenotypes, thus forming pre-metastatic inflammatory niches [45].

#### Stromal cells

Through the regulation of stromal cells in secondary organs, the microenvironment of metastatic cancer is formed, and the immune response, inflammation, angiogenesis, matrix remodelling and organ tendency of cancer cell metastasis are regulated [46]. Song et al. confirmed that the peritoneal macrophages are closely related to peritoneal metastasis of gastric cancer. In detail, peritoneal macrophages support angiogenesis and cancer growth through the production of vascular endothelial growth factor (VEGF) and epidermal growth factor (EGF) [47]. Similarly, CAFs-derived hydrogen peroxide-induced clone 5 (HIC-5) regulates cytokines and modifies the ECM to regulate the invasion of oesophageal squamous cell carcinoma (ESCC) cells [48].

#### Epithelial-mesenchymal transition

The course of epithelial-mesenchymal transition (EMT) promotes the spread of cancer [49]. Macrophages, platelets and mesenchymal stem cells (MSCs) can promote the EMT, separate cancer cells from contact with neighbouring epithelial cells and acquire motor/invasive phenotypes [26]. The basement membrane and the stroma are degraded and invaded by the EMT, which can cause cancer cells to enter circulation through lymphatic or blood pathways, secrete growth factors and cytokines, and survive under the protective interaction between them and platelets [50]. When the cancer cells remain in the narrow capillaries of the target organs, the cancer cells can destroy the endothelial cell connection, infiltrate the surrounding tissue, and maintain the initial dormant state until the conditions are conducive to the colonization of metastasis [51]. In addition, the EMT can promote the differentiation of cancer cells into cancer stem cells [52]. In the TME, immune cells and stromal cells, such as TA-MSCs and CAFs, are considered to constitute the microenvironment of cancer stem cells, which regulate the fate of cancer stem cells by providing signals composed of cell-cell contact and the secretion of factors (growth factors and cytokines that promote CSC self-renewal) [53].Foe example, STAT3 is induced by IL-6 and other inflammatory factors [54]. Moreover, the interaction between cancer stem cells and the TME promotes cancer progression; for example, glioma stem cells (GSCs) can preferentially secrete Wnt-induced signal protein 1 (WISP1) and promote the development of the TME by promoting the survival of GSCs and TAMs through WISP1 [55]. In addition, in all types of cancers, CSCs showed changes in energy balance and metabolic status, such as enhanced glycolysis, compared with non-CSCs [56]. Therefore, in the TME, the metabolic changes induced by inflammation may be involved in the formation of CSCs and carcinogenesis.

#### Promotion factors or stromal components

The pre-metastatic niche is established and initiated via the intricate interaction among local matrix components, primary cancer-derived factors and cancer-mobilized bone marrow-derived cells [5]. For example, in a mouse model of lung metastasis, MDSCs are a key factor in the formation of the microenvironment before metastasis after the resection of the primary cancer [57]. More specifically, the pre-metastatic niche is established by recruited bone marrow-derived cells (BMDCs) and immune cells. These cells can secrete growth factors, inflammatory cytokines and angiogenic molecules to reshape the local microenvironment and support cancer cell invasion, colonization and proliferation [58].

#### Extracellular matrix

Treg cells, cancer-associated neutrophils, MDSCs and TAMs can be recruited to secondary organs by cancer cell-derived cytokines and chemokines, and facilitate metastasis by supporting the development of pre-metastatic niches [59]. CXCR4/TGF-β1 can boost the liver metastasis of colon cancer by mediating the differentiation of HSCs into CAFs [60].

In addition, in the secondary sites of breast cancer (lungs), lysyl oxidase, periostin and tenascin C, together with the pro-metastatic molecules Coco and N-acetylgalactosamine transferase 14 (GALNT14), promote lung metastatic cell colonization and ECM remodelling, resulting in the formation of a pre-metastatic niche (PMN) [29]. Du et al. discovered that the combination of mesenchymal stem cell-derived interleukin (IL)-8 and C-X-C chemokine receptor (CXCR)-1 promotes osteosarcoma cell anoikis resistance and lung metastasis by activating the Akt signalling pathway [61]. The hypoxic state of the TME increases the recruitment of Tregs by inducing the expression of chemokine CC-chemokine ligand 28 (CCL28), which promotes the immunosuppression of the TME [62]. In addition, cancer hypoxia hinders the function of MDSCs in the TME through HIF-1α and turnstheir differentiation state into TAMs [63].

#### Adenosine-triphosphate

The change of adenosine triphosphate in the PMN is also a key point of cancer metastasis. Li et al. confirmed that one of the reasons for the promotion of the metastasis of cancer cells is the higher concentration of extracellular ATP in cancer tissue than in normal tissue [64]. The main mechanism by which extracellular ATP (eATP) promotes metastasis is to increase the concentration of intracellular free Ca^2+^, which promotes the release of several cytokines and triggers the EMT [65].

### Metastatic microenvironment

Cancer stem cells and cancer cells exudate from the primary focus, infiltrate into the extracellular matrix, and promote angiogenesis or cell infiltration into the circulatory system, thus evading the host’s defence mechanism through chemotaxis, causing it to migrate to specific vascular sites, adhere, and then exudate blood vessels and return to a specific environment until metastatic foci are formed (Fig. 1). Metastatic cancer cells usually reside in distal tissues and organs in a dormant state. The mechanism of controlling metastatic dormancy includes the regulation of the expression of genes in disseminated cancer cells (DTCs), including genetic and/or epigenetic control, as well as the regulation mechanism of the TME [66]. For example, the niche after liver metastasis, which develops after cancer cells enter the liver, can be divided into four key stages (i) microvascular, (ii) preangiogenesis, (iii) angiogenesis and (iv) growth stages [67].

#### Stromal cells and the extracellular matrix

Immunosuppressive cells are recruited into cancer to help establish a state of immunosuppression in secondary tissues. Treg cells and MDSCs secrete anti-inflammatory cytokines, which inhibit the anti-cancer ability of immune cells. Once micrometastases overcome their dormancy, they receive signals and cells from the microenvironment to further support their invasion. For example, the coagulation system and components of platelets, such as tissue factor (TF), are crucial mediators of metastatic growth, which can interfere with NK cells to undermine micrometastases or support clot formation, leading to the recruitment of MDSCs [26]. In the lung metastasis of breast cancer, IL-1α and IL-1β secreted by breast cancer cells induce the production of CXCL9 and CXCL10 in lung fibroblasts through NF-κB signal transduction, thus promoting the growth of lung metastasis [68]. Li et al. found that CAFs-derived lysyl oxidase (LOX) in liver metastases of gastric cancer promotes niche formation and growth, indicating a poor prognosis. In the meantime, cancer cells secrete transforming growth factor-β-1 to nourish CAFs and stimulate them to produce more lysyl oxidase [69]. In addition, the metabolism of CAFs also undergoes fundamental changes during the activation process. For example, CAFs use aerobic glycolysis to maintain the enhanced proliferative activity of cancer cells rather than relying on oxidative phosphorylation (OXPHOS) [70]. CAFs use carbon from different sources to produce glutamine for cancer cells to promote ovarian cancer progression [71]. CAFs also play important roles in drug resistance. CAFs can cause drug resistance by providing a protective environment for cancer cells [72]. The cancer interstitial pressure it produces also limits the entry of drugs into cancer cells and indirectly induces drug resistance [73].

#### Characteristics of metastatic organs

The microenvironments of different metastatic organs of cancer have their own characteristics. Kaplan et al. demonstrated that resident fibroblasts in secondary organs can help primary cancer upregulate the expression of fibronectin. These fibroblasts are also the junction of the VEGFR1^+^ haematopoietic progenitor cell (HPC) cluster and migratory cancer cells [74]. Further research on the role of secretory factors in maintaining and overcoming dormancy comes from screening metastatic breast cancer. Interestingly, Gao et al. showed that some signals work only in the lungs not in the bone and brain, suggesting that metastasis-initiating cells can cover microenvironment-mediated inhibition in an organ-specific way [75]. On the other hand, Yoshikawa et al. found that M2-polarized macrophages in peripheral blood are related to the construction and growth of liver metastases in patients with pancreatic cancer [76]. Therefore, the special characteristics of the microenvironment of metastatic organs are also a valuable research direction.

### Mechanism of drug resistance mediated by TME

The TME is also a protective barrier for cancers. The interaction between cancer cells and the TME enables some cancer cells to escape apoptosis and develop drug resistance. Microenvironment-mediated drug resistance may be caused by the adhesion of cancer cells to interstitial fibroblasts or extracellular matrix components, or by soluble factors secreted by stromal cells, or mediated by the immune response [77]. For example, stromal cells from lymph nodes promote resistance to 5-fluorouracil and oxaliplatin through SDF1/CXCR4-dependent mechanisms [78]. Studies on melanoma have also confirmed that CAFs promote the metastasis and drug resistance of melanoma cells by increasing the expression of MMP1 and MMP2 [79]. Gemcitabine and 5-fluorouracil can activate MDSCs, and induce the production of IL-1β, which induces the Th17 response and weakens the anticancer effect [80]. Therefore, taking the TME as the target may destroy the interaction between the TME and cancer cells and make follow-up treatment more effective.

### Liquid TME

The liquid TME is mainly formed by the interaction between haematopoietic cancer cells and stromal cells, such as B-cell lymphoma, including follicular lymphoma (FL), mantle cell lymphoma (MCL), chronic lymphocytic leukaemia (CLL), classical Hodgkin’s lymphoma (CHL) and mucosa-associated lymphoid tissue lymphoma (MALT), providing striking examples of a pivotal interaction of haematopoietic cancer cells with stromal cells [81]. The important components of its microenvironment are monocyte-derived milk cells (NLCs), mesenchymal stromal cells, T cells and NK cells, which communicate with cancer cells through a complex network of adhesion molecules, chemokine receptors, cancer necrosis factor (TNF) family members and soluble factors [82]. Unlike solid cancers, some of these cell types and precursors were already present in secondary lymphoid organs before the onset of lymphoma [83]. For example, the specialized fibroblast reticular cells (FRCs) that make up the trunk of SLOs are essential for organ development and the division of T and B cell regions and participate in the acquired immune response [84]. There is a great difference between liquid cancers and solid cancers, and the mechanisms of their microenvironment and components are very different. For example, there is a detailed understanding of TAMs in solid cancers, but the mechanism of TAMs in liquid cancers is not well understood [85]. Through the study of the solid TME, we can provide some new ideas for the study of the liquid TME.

## Microenvironment-specific interventions

Currently, the TME is the focus of cancer metastasis and growth research. Many intervention strategies are applied to change the TME, including chemotherapy, targeted therapy, immunotherapy, nanoparticle systems, traditional Chinese medicine, and combined drug therapy (see Table 1). Moreover, many drugs for the treatment of the TME (monotherapy or combination therapy) have entered the clinical trial stage and are actively recruiting patients (Table 2).

**Table 1:** Comprehensive list of drugs for the TME and their mechanisms of action

Drug	Inhibitory mechanisms	Mode of action	Test mode	Reference
Oxaliplatin	Immunosuppressive cells	Increase activation of CD8 + T cells, reduce cancer CD11b + F4/80 high macrophages, and reduce spleen MDSCs	In vitro and in vivo test	[86]
PG545	Growth factor-mediated cell invasion	Reduces the phosphorylation of AKT, EGFR and ERK induced by HB-EGF	Phase I clinical trials	[87]
Gemcitabine	EMT	Reduce the frequency of CTC and the logarithm of CTC	In vitro and in vivo test	[88]
Paclitaxel	EMT	Reduce the frequency of CTC and the logarithm of CTC	In vitro and in vivo test	[88]
Fludarabine	Brain cancer cells	X-inactivated specific transcript	In vitro and in vivo test	[89]
Rapamycin	TAMs	Reduction in the expression of Bcl-2 and Survivin and an increase in the expression of Smac	In vitro test	[90]
Apatinib	EMT/Angiogenesis	Targeting STAT3/block PI3K/AKT and VEGFR2/RAF/MEK/ERK signaling pathways	In vitro and in vivo test	[91, 92]
WRG-28	Cancer invasion and migration	DDR2	In vitro and in vivo test	[93]
Bortezomib	CAF	Caspase-3	In vitro and in vivo test	[94]
Pambarbital	CAF	Caspase-3	In vitro and in vivo test	[94]
Apelin inhibitor	Angiogenesis/MDSCs	Apelin	In vitro and in vivo test	[95]
Dasatinib	TAMS	Inhibited the self-renewal ability of H460R and A549R cells	In vitro test	[96]
Repagenil	Cancer cell	MSCs	In vitro and in vivo test	[97]
Anti-CTLA-4 antibody	T cells	Enhance antibody-dependent cell-mediated cytotoxicity, phagocytosis	Preclinical trial	[98]
Transforming growth factor-β inhibitors	Cancer cell/releasing cytotoxic T cells/promote T cell infiltration	Transforming growth factor-β	In vitro and in vivo test	[99, 100]
Plerixafor	Angiogenesis	CXCR4	In vitro and in vivo test	[101]
Macrophage receptor with collagen structure	Cancer proliferation	E-programming of macrophages	In vitro and in vivo test	[102]
Embeline	Growth of pancreatic cancer	Increasing the infiltration of Th1 cells, NK, CTL, γ δ T and NKT, and reducing the infiltration of Th17, PMN-MDSC, IL-8 and IL-6 positive immune cells	In vitro and in vivo test	[103]
Functionalized micellar	Reverse the abnormal expression of several key marker proteins	Inhibit the adhesion of activated endothelial cells to circulating cancer cell	In vitro and in vivo test	[104]
Cancer matrix-targeted nano-carrier	Cut off the support of the matrix to cancer cells	Remove CAFs,	In vitro and in vivo test	[105]
Nanoparticles-based photoimmunotherapy	T cells	CAFs	In vitro and in vivo test	[106]
Curcumin	Cancer cells, Angiogenesis	VEGF, IL-6 and cancer stem cells, transcription factor nuclear factor-NB (NF-NB), signal transduction, transcriptional activator 3 and angiogenic cytokines	In vitro and in vivo test	[107, 108]
APG-157	Attract immune cells into the TME	Increased expression of CD4+ and CD8+ cells and increased expression of PD-1 and PD-L1	Phase I placebo controlled trial	[109]
Sophoridine	Macrophage	TLR4/IRF3 pathway	In vitro test	[110]
Ginsenoside Rh2	Improve TME	Regulating the phenotype of TAMs	In vitro and in vivo test	[111]
Berberine	EMT	Smad-independent and Smad-dependent transforming growth factor-β signaling pathway	In vitro test	[112]
Wogonin	EMT	IL-6/STAT3 signal pathway	In vitro and in vivo test	[113]
Bigelovin	EMT	N-and E-cadherin, STAT3 pathway, and cofilin pathway	In vitro and in vivo test	[114]
Cordycepin	Up-regulating cancer cell apoptosis and eliciting cell cycle arrest	CSCs	In vitro test	[115]
Shikonin	Cancer cell	Exosome	In vitro test	[116]
6-gingerol (6G)	TME	Promoting cancer vascular normalization, reducing microvascular structure entropy (MSE)	In vitro and in vivo test	[117]
Salvianolic acid A	Angiogenesis	Block the secretion of glucose-regulated protein 78	In vitro and in vivo test	[118]
Dihydrodiosgenin	Inhibit HCC metastasis	Inhibit platelet activation and reduce endothelial cell-derived factor VIII	In vitro and in vivo test	[119]
Poly (adenosine diphosphate–ribose) polymerase (PARP) inhibitor (PARPI)	Up-regulate PD-L1	Promoting the activation of IFN pathway, Up-regulate PD-L1	Preclinical trial	[120, 121]
The combination of PARPI and mitogen-activated protein kinase (MEK) inhibitor	TME	Induces BIM-mediated apoptosis by activating caspase-3, inhibits the expression of CD31 in endothelial cells, and inhibits the production of mutant RAS-induced VEGF through RAS/MAPK pathway	In vitro and in vivo test	[122]
Sorafenib combined with bufalin	Angiogenesis	mTOR/VEGF signal pathway	In vitro and in vivo test	[123]
Ginsenoside Rg3 combined with cisplatin	TME	EMT	In vitro and in vivo test	[124]

**Table 2:** Selective Actively Recruiting Clinical Trials for patients

Name of study Clinical	Phase	Conditions	Therapy	Measure	Clinicaltrials.gov Identifier
A Study of ALKS 4230 on the TME	Phase 2	Advanced Solid cancer	ALKS 4230 + pembrolizumab	Total T cells, CD8^+^ T cells, CD56^+^ cells and Treg cells	NCT04592653
Effects of MK-3475 (Pembrolizumab) on the Breast TME in Triple Negative Breast Cancer		Triple Negative Breast Cancer	Merck 3475 Pembrolizumab	Number of subjects with significant mean percent change in TILs	NCT02977468
Analysis of the Modulation of the TME by MK-3475 (Pembrolizumab) Using a Systems Biology Approach (PEMSYS)	Phase 2	Metastatic Melanoma Naive to Immune Therapy in Metastatic Setting	Pembrolizumab - additional treatment	Bioinformatics	NCT03534635
GVAX Pancreas Vaccine (With CY) in Combination With Nivolumab and SBRT for Patients With Borderline Resectable Pancreatic Cancer	Phase 2	Pancreatic Cancer	Cyclophosphamide	CD8 count (cells/mm^3) in the TME	NCT03161379
L-DOS47 Plus Doxorubicin in Advanced Pancreatic Cancer	Phase 1/Phase 2	Pancreas Cancer	L-DOS47 Plus Doxorubicin	Cancer pH, Proportion of patients expressing anti-L-DOS47 antibodies	NCT04203641

### Chemotherapy drugs for the TME

**Fig. 2:**
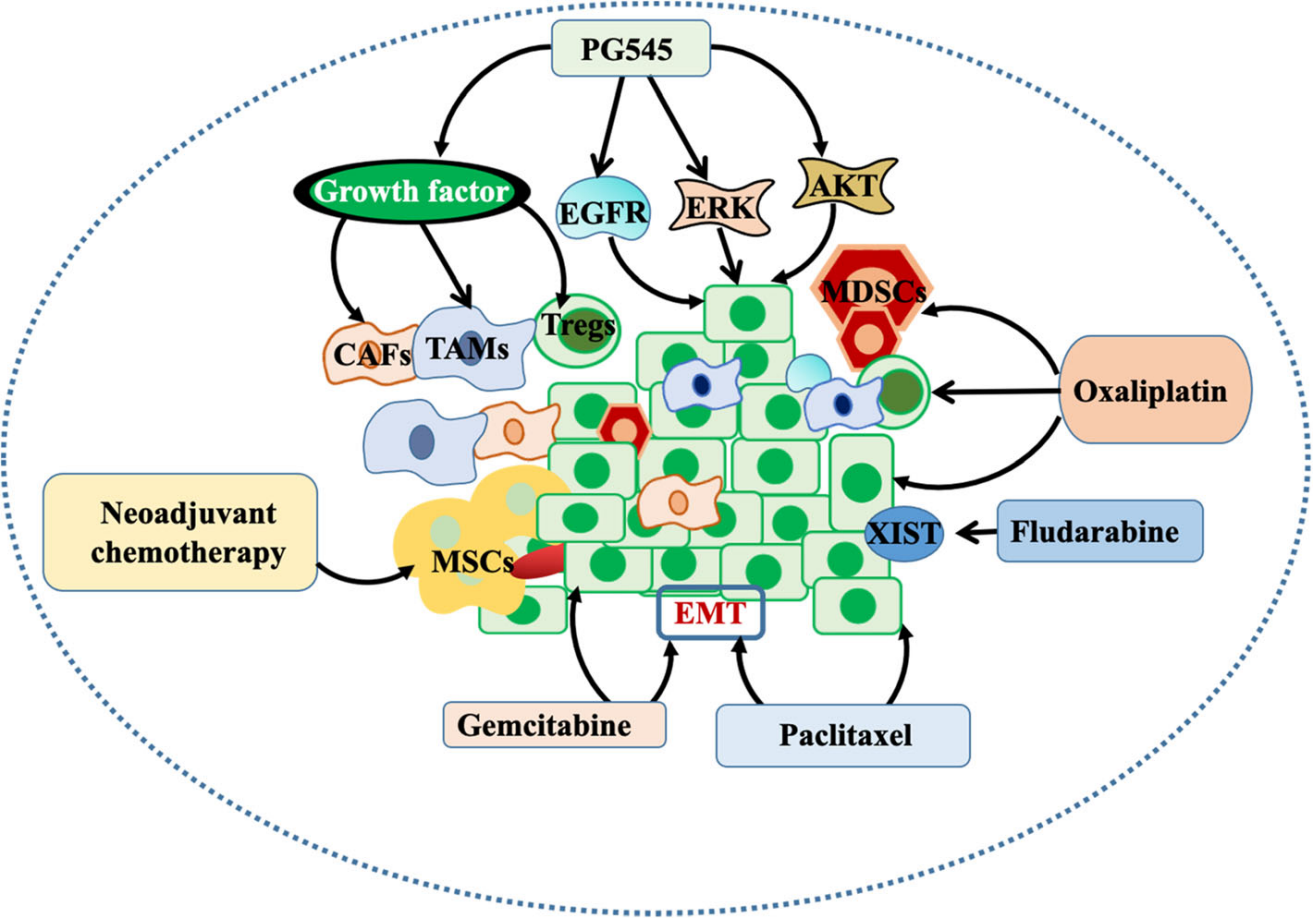
Intervention of chemotherapy on the TME. Neoadjuvant chemotherapy can increase the density of myeloid suppressor cells. Oxaliplatin (OXP) can increase cancer infiltration and activation of CD8^+^ T cells and reduce cancer CD11b^+^F4/80^high^ macrophages and spleen MDSCs. PG545 inhibits growth factor-mediated cell invasion, reduces the HB-EGF-induced phosphorylation of AKT, EGFR and ERK, and reduces the cancer burden. Gemcitabine (GEM) or paclitaxel (PTX) inhibited the EMT by reducing the frequency of CTCs and the logarithm of CTCs. Fludarabine has high selectivity for cells with low expression of X-inactivated specific transcription (XIST) and inhibits the growth of brain cancer cells

Chemotherapeutic drugs, also known as cytotoxic drugs, usually have anti-cancer effects by acting on key cellular biological events necessary for cancer cell survival and proliferation. Their effects on various components in the TME have become a research point (Fig. 2).

Chemotherapy can activate the local immune state, which regulates the anti-cancer T cell response by enhancing the effector T cell response, disrupting the immunosuppressive pathway and increasing cancer antigenicity [86–88]. By comparing the therapeutic effects of 27 patients with osteosarcoma before and after treatment, neoadjuvant chemotherapy was related to an the increase in the density of CD8^+^ T cells, CD3^+^ T cells, Ki67^+^CD8^+^ T cells and PD-L1^+^ immune cells, and the myeloid suppressor cells of HLA-DR-CD33^+^ decreased significantly after treatment [89]. Oxaliplatin (OXP) alone can eliminate immunosuppressive cells, suppress cancer growth and induce an anti-cancer immunostimulatory microenvironment. For example, for the model of the abdominal metastasis of colon cancer, the administration of OXP can increase cancer infiltration and activation of CD8^+^ T cells, reduce cancer CD11b^+^F4/80^high^ macrophages, and reduce spleen MDSCs, thus affecting the cancer immune microenvironment [125].

Through preclinical studies and phase I clinical trials of four patients with advanced solid cancers (thyroid cancer, colon cancer, pancreatic cancer and melanoma), Winterhoff et al. found that PG545 suppresses growth factor-mediated cell invasion; reduces the phosphorylation of AKT, EGFR and ERK induced by HB-EGF; and significantly abates the cancer burden, which is strengthened when combined with carboplatin in the SKOV-3 model or paclitaxel in the A2780 model [90]. In a mouse model of pancreatic ductal adenocarcinoma (PDAC), gemcitabine (GEM) or paclitaxel (PTX) could inhibit EMT, to reduce the frequency of CTCs and the logarithm of CTCs in circulating cancer cells, thus reducing cancer metastasis [91].

There is no doubt about the status of chemotherapy as the earliest treatment for cancer. In the model of brain metastasis of breast cancer, fludarabine is highly selective for cells with low expression of X-inactivated specific transcript (XIST), which significantly inhibits the growth of brain cancer cells, delays the occurrence of brain metastasis, and has no obvious toxicity [92]. Chemotherapy has its own advantages, but patients have poor tolerance and strong drug resistance. It may affect the TME to promote the spread of cancer cells to secondary sites [93]. Therefore, how to reduce the side effects of chemotherapy in microenvironment treatment in clinical practice may be of great concern.

### Targeted drugs for the TME

**Fig. 3:**
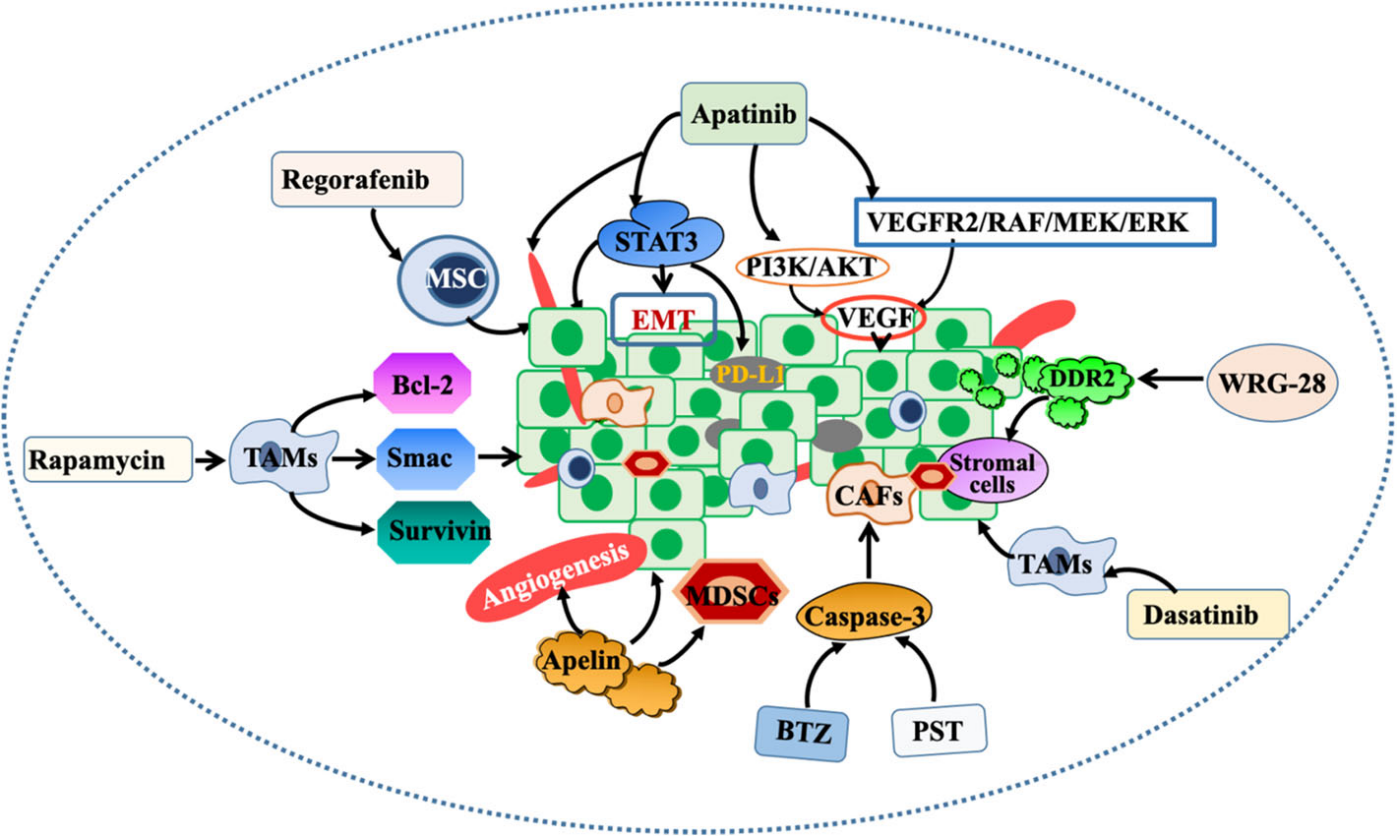
Intervention of targeted therapy on the TME. Regorafenib inhibits the interaction between mesenchymal stem cells (MSCs) and cancer cells. Rapamycin-mediated autophagy can reduce the expression of Bcl-2 and survivin and increase the expression of Smac in TAMS. Apatinib can reduce cancer angiogenesis and inhibit the expression of PD-L1 through targeted STAT3 inhibition of the EMT and blockade of the PI3K/AKT and VEGFR2/RAF/MEK/ERK signalling pathways, thus affecting VEGF-mediated cell proliferation and invasion. WRG-28 inhibits cancer invasion and migration by targeting DDR2. Dasatinib reduced the M2 polarization of TAMS. Bortezomib (BTZ) and phenobarbital (PST) can reduce the survival rate of CAFs and inhibit the proliferation of cancer cells by inducing caspase-3-mediated apoptosis. The inhibition of apelin can inhibit angiogenesis and growth, and reduce the infiltration of suppressor cells derived from the polymorphonuclear myeloid system

The TME is complex and diverse, in which various components and characteristics are closely related to cancer occurrence and development, and thus, targeted regulation of components or signalling pathways in the TME has become the key to suppressing cancer proliferation and invasion (Fig. 3). For instance, Shao et al. confirmed that rapamycin-mediated autophagy can result in a reduction in the expression of Bcl-2 and survivin and an increase in the expression of Smac in TAMs. The upregulation of TAM autophagy inhibits the propagation of colon cancer cells, induces apoptosis, and changes the expression of radiosensitivity-related proteins [94].

The same drug may have an effect on different targets in the microenvironment of different types of cancers. For example, in a mouse model of human hepatocellular carcinoma xenotransplantation, apatinib can effectively reduce cancer angiogenesis, inhibit cancer growth and prolong animal survival [95]. In an osteosarcoma model, apatinib suppresses the invasion and migration of cancer cells and the expression of PD-L1 by targeting STAT3 to inhibit the EMT. Interestingly, it can also block the PI3K/AKT and VEGFR2/RAF/MEK/ERK signalling pathways in cholangiocarcinoma cells, thus affecting VEGF-mediated cell proliferation and invasion [126]. This suggests that the existing targeted drugs may have greater potential and are worthy of our study.

Different targets in the microenvironment of the same cancer type are often affected by different drugs to inhibit cancer proliferation and metastasis. In experimental mouse models of breast cancer, WRG-28 inhibits cancer invasion and migration by targeting DDR2, reduces the supporting effect of the matrix on cancer, and thus inhibits the colonization of metastatic breast cancer cells in the lungs [127]. Lee et al. screened 51 drugs that are in clinical trials or approved by the FDA and found that bortezomib (BTZ) and phenobarbital (PST) can reduce the survival rate of CAFs by inducing caspase-3-mediated apoptosis and inhibit the proliferation of cancer cells in a breast cancer mouse transplantation model [96]. In addition, Uriesalgo et al. showed that targeting apelin with apelin inhibitors can inhibit angiogenesis and growth in breast and lung cancer models without increasing TME hypoxia, improve vascular function, and reduce the infiltration of polymorphonuclear myeloid derived suppressor cells [97]. Therefore, the synergistic effect of different targeted drugs on the TME can be considered for the prevention and treatment of cancer.

Therapeutic and targeted delivery at the cancer site can be achieved by modifying exosomes with corresponding targeting ligands, for example, in mouse models of breast and ovarian cancer, DOX enhances its targeting through exosomes and effectively inhibits cancer progression [128]. The discovery of an antibody-functionalized exosome-targeting delivery system may be a new human cancer drug delivery system. In vivo experiments have shown that the A33 antibody-functionalized exocrine targeted delivery of doxorubicin can inhibit the growth of colorectal cancers, prolong the survival time of mice and reduce cardiotoxicity [129].

Similarly, for cisplatin-resistant non-small-cell lung cancer, dasatinib inhibited the self-renewal ability of H460R and A549R cells and reduced the M2 polarization of TAMS [130]. Takigawa et al. suggested that regorafenib affects the interaction between MSCs and cancer cells by targeting the TME, thus inhibiting the proliferation and migration of colon cancers in mice [98]. In a randomized, placebo-controlled phase II clinical trial, tasquiimod, a new oral targeted therapy for the TME, increased progression-free survival (PFS) in prostate cancer patients with (MCRPC) metastatic castration resistance [131]. Targeted therapy is a part of precision therapy, but for some cancers, its curative effect is not obvious, and there are some limitations and side effects. Therefore, while exploring potential targets and targeted drugs, it may be more important to apply the existing research results to the clinical setting and investigate how to better cooperate with other treatment methods to enhance the therapeutic effect.

### Immunotherapy drugs for the TME

**Fig. 4:**
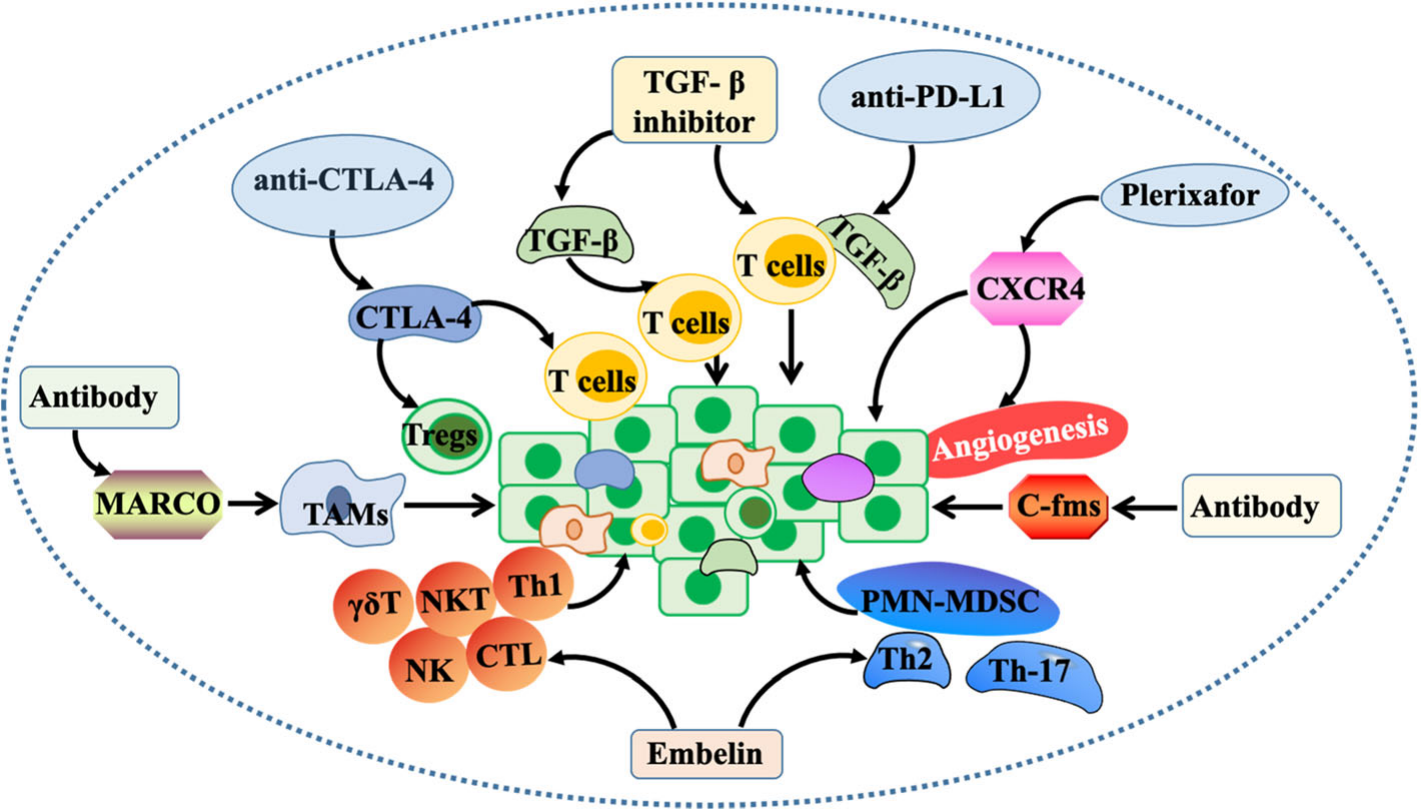
Intervention of immunotherapy on the TME. Anti-CTLA-4 antibody consumes regulatory T cells and removes Tregs. The use of transforming growth factor-β (TGF-β) inhibitors and anti-PD-L1 antibodies can reduce the TGF-β signal and promote the infiltration of T cells into the cancer centre. Plerixafor inhibits CXCR4 and reduces cancer spread and angiogenesis. Embelin can regulate the cancer-immune microenvironment by increasing the infiltration of Th1 cells, NK cells, CTLs, γδT cells and NKT cells, reducing the infiltration of Th17, PMN-MDSCs, and IL-8- and IL-6-positive immune cells. Anti-c-FMS antibody affects the establishment of breast cancer cells in bone

Cancer immunotherapy is a type of immune function that stimulates or removes immunosuppression as a cancer treatment strategy to effectively inhibit cancer development. It produces immune memory, effectively restrains the resistance of malignant cancer to prevent the proliferation of cancer recurrence, restarts and maintains cancer-immune circulation, and restores the body’s normal anticancer immune responses. The goal of cancer immunotherapy is to initiate a self-sustaining cancer immune cycle that can self-amplify and spread while minimizing the self-inflammation associated with treatment [132] (Fig. 4).

Immunotherapy has a unique mechanism of action on stromal cells. Clinically, cytotoxic T lymphocyte associated protein 4 (CTLA-4) and programmed cell death protein 1 (PD-1) are two checkpoints that can be successfully targeted [99]. Zhang et al. developed a new generation of anti-CTLA-4 antibodies. Preclinical studies have proven that they selectively consume regulatory T cells in the TME to enhance antibody-dependent cell-mediated cytotoxicity/phagocytosis (ADCC/ADCP) and reduce immunotherapy-related adverse events (IRAEs) [100]. The preservation of CTLA-4 checkpoints may be more effective in removing Tregs from the TME, thus improving the efficacy [101].

On the other hand, anti-PD-L1/PD-1 antibodies can restore T cell immunity by interfering with the PD-L1/PD-1 pathway, which leads to lasting remission in some cancer patients [102]. Tauriello et al. found the inhibition of liver metastasis of colon cancer by transforming growth factor-β in mice, thus releasing cytotoxic T cells to respond to cancer cells to prevent metastasis, and thus, the use of TGF-β inhibitors to achieve immuno-osmosis is sufficient to increase the sensitivity to anti-PD-1/PD-L1 checkpoint therapy [103]. It is not surprising that the combination of TGF-β inhibitors and anti-PD-L1 antibodies can reduce the TGF-β signal in stromal cells, promote T cell infiltration to the cancer centre, and stimulate anti-cancer immunity and cancer regression [133]. In the preclinical model of prostate cancer, the inhibition of CXCR4 by plerixafor can reduce invasiveness in vitro, which in turn reduces cancer spread and related angiogenesis [134].

In addition, Georgoudaki et al. found that in breast and colon cancer as well as melanoma models, immunotherapy with “macrophage receptor with collagen structure” (MARCO) can prevent cancer proliferation and migration and improve the immunogenicity of the TME. E-programming of macrophages in the TME with monoclonal antibodies is a feasible method for cancer immunotherapy [135]. Marsh et al. found that embelin can inhibit the growth of pancreatic cancer in KrasG12D mice by increasing the infiltration of Th1 cells, NK cells, CTLs, γδT cells and NKT cells, and reducing the infiltration of Th17, PMN-MDSC, and IL-8- and IL-6-positive immune cells, thus regulating the cancer-immune microenvironment [136]. Jeffery et al. proved for the first time that anti-c-FMS antibody affects the establishment of breast cancer cells in bone through a mouse model of breast cancer [137].

Immunotherapy is changing the treatment of solid cancers, and current clinical work is focused on developing immunotherapy combinations to transform non-responders into responders, deepen their responses, and overcome their drug resistance. Therefore, the identification of immune markers can predict the response potential of immunotherapy and determine the best combination of immunotherapy for specific patients to carry out effective immunotherapy for cancer patients [138]. For example, human epidermal growth factor receptor-2^+^ (HER-2^+^) breast cancer and TNBC are more likely to have interstitial infiltrating immune cells (TILs), than luminal breast cancer, and there is a linear relationship between the TILs content and clinical results. The possibility of expressing programmed death ligand-1 (PD-L1) in the TME is also higher than that in luminal breast cancer [139–141].

Progression-free survival and overall survival were longer in stage III NSCLC patients undergoing chemoradiotherapy when the density of CD8+ cancer-infiltrating lymphocytes in the pre-treated biopsy specimens was higher than that in patients with low CD8+ cancer infiltrating lymphocyte density [142]. This is through the immune mechanism to improve disease control, and thus, the baseline cancer-infiltrating lymphocyte status can be used as a predictive biomarker of checkpoint inhibitory immunotherapy and as a prognostic biomarker. There may also be a variety of immunosuppressive mechanisms in the TME, including CTLA-4, PD-L2 and interleukins [143]. Strategies that combine multiple approaches to detect the immune status of the TME may be more effective in treating immune checkpoint inhibitors [144].

As a new treatment, immunotherapy is not aimed at tissues and cancer cells but at the body’s own immune system to change the immune state of the TME and prevent and treat cancer progression. However, although the therapeutic effect is good, the proportion of the effective population is low. Therefore, the establishment of predictive biomarkers of checkpoint immunotherapy is very important to maximize the effectiveness of treatment. For patients for whom immunotherapy is ineffective at the current checkpoint, unnecessary toxicity can be avoided in time, and alternative treatment strategies can be adopted. Although immunotherapy research is in a bottleneck at present, it has broad application prospects in the clinical setting.

### Nanoparticle system intervention for the TME

**Fig. 5:**
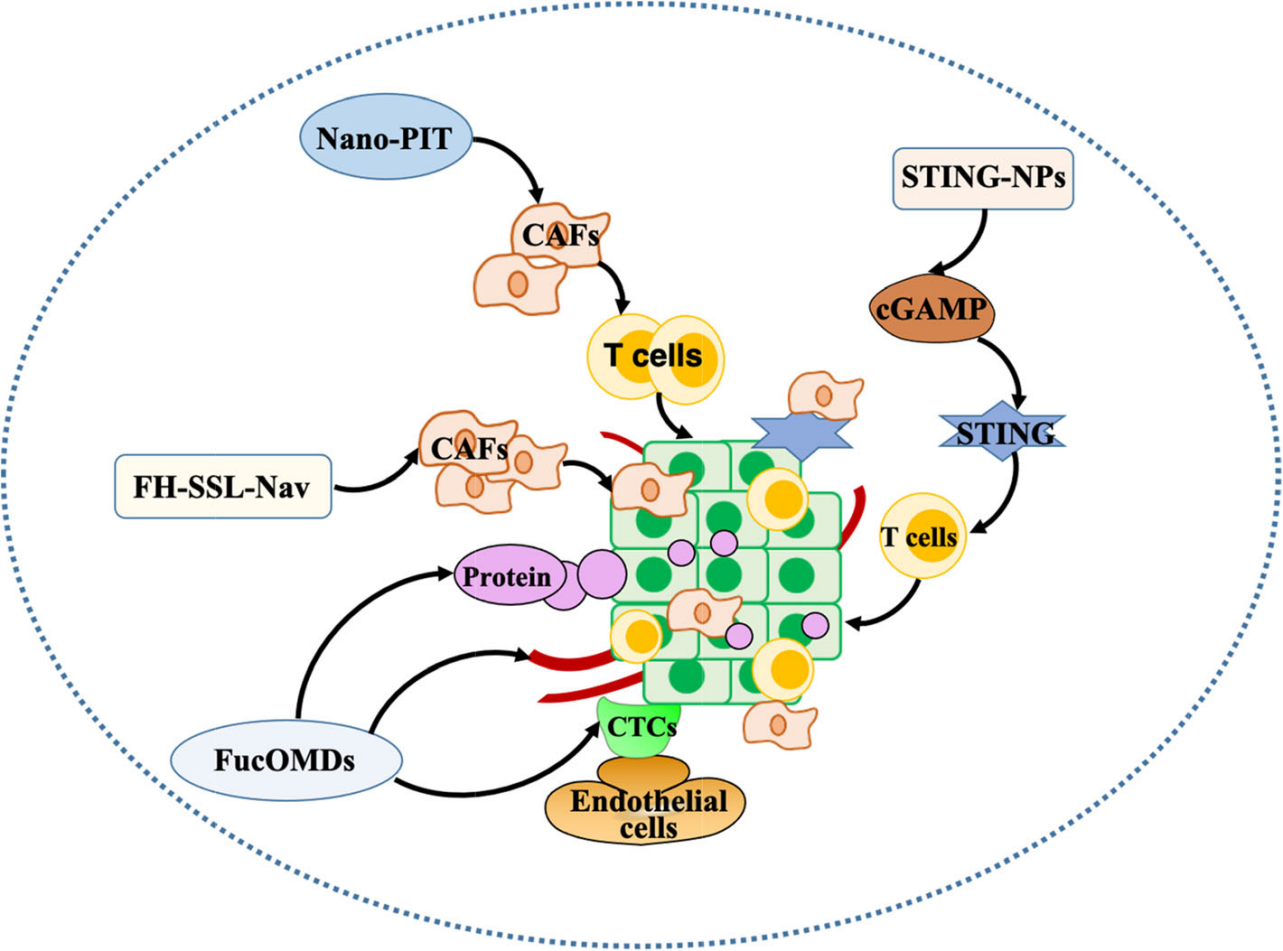
Intervention of the nanoparticle-based drug delivery system in the TME. Functionalized micellar endothelial cells (FucOMDs) adhere to cancer cells and reverse the abnormal expression of several key marker proteins in the pre-metastatic niche. A new type of cancer matrix-targeted nanocarrier (FH-SSL-Nav) can remove CAFs. Photoimmunotherapy (nano-PIT) selectively kills CAFs and increases the invasiveness of T cells. Interferon gene-activated nanoparticle stimulator (STINNP) enhances the cytoplasmic delivery of cyclic guanosine monophosphate-adenosine monophosphate (CGAMP) through an in vivo escape mechanism, activating STING and triggering T cells

The use of nanocarriers not only increases the bioavailability and solubility of hydrophobic drugs but also improves the pharmacokinetic properties of drugs, avoids the degradation of drugs in vivo circulation, and delivers one or more drugs to the lesion site to achieve the controlled release of drugs. Nanotechnology helps to improve the targeting efficiency, has received increasing attention, and has been used in a variety of treatments, such as chemotherapy, radiotherapy and immunotherapy [145] (Fig. 5).

In recent years, stimulus-sensitive or intelligent nanocarriers have attracted much attention because of their advantages in controlling the release of drugs. By replying to external stimuli (e.g., ultrasound and magnetism) or internal stimuli (e.g., temperature, pH, and H_2_O_2_), medicine can be controllably released from smart nanocarriers at the therapeutic target in a desired manner [146–148].

Nanocarriers can improve the solubility of drugs, increase the stability of drugs, reduce toxicity and side effects, and specifically deliver medicinal molecules to cancers by heightened targeting effects or retention (EPR) effects and permeability [149, 150]. Moreover, nanocarriers can encapsulate various anticancer drugs with different treatment mechanisms and deliver them at the same time; therefore, they are very beneficial to the combined therapy of cancers [104, 105]. Zhang et al. demonstrated through in vivo experiments that targeted nanoparticles can remove pro-cancer cells and stimulate anti-cancer effector cells by loading immunomodulators (such as lipid nanoparticles coated with cancer-targeting peptides IRGD and PI3K inhibitors) [106]. This not only helps to reduce the toxicity and side effects of antineoplastic medicine, but also helps to exert the congenerous effect of different antineoplastic medicines [151].

In addition to the above effects, the material of the nanoparticles themselves also has a direct impact on the TME. Jiang et al. found that functionalized micelles (FucOMDs) target and have good blood circulation, which can inhibit the adhesion of activated endothelial cells to CTCs in triple-negative breast cancer mice and reverse the abnormal expression of several key marker proteins in the pre-metastatic niche [107]. Chen et al. discovered that a new type of cancer matrix-targeted nanocarrier (FH-SSL-Nav) can specifically remove CAFs from the liver cancer model to promote the penetration of nanodrugs into the cancer and cut off the support of the matrix to cancer cells [108]. In addition, Zhen and others also put forward their own point of view, indicating that the selective killing of CAFs and nanoparticle-based photoimmunotherapy (nano-PIT) can increase the invasiveness of T cells, thus effectively inhibiting cancer, which has also been proven by in vivo experiments [109].

At present, research on nanoparticle systems is mainly focused on accurate drug delivery, precise controlled release, and the efficacy of the material itself. As an ideal adjuvant therapy, there is no denying its advantages and bright prospects in clinical application. However, everything has two sides, and we do not have a deep understanding of it nor are we very clear about its side effects. Therefore,we should evaluate it more comprehensively and examine it before it is used in the clinical setting.

### Natural products from traditional Chinese medicine for the TME

**Fig. 6:**
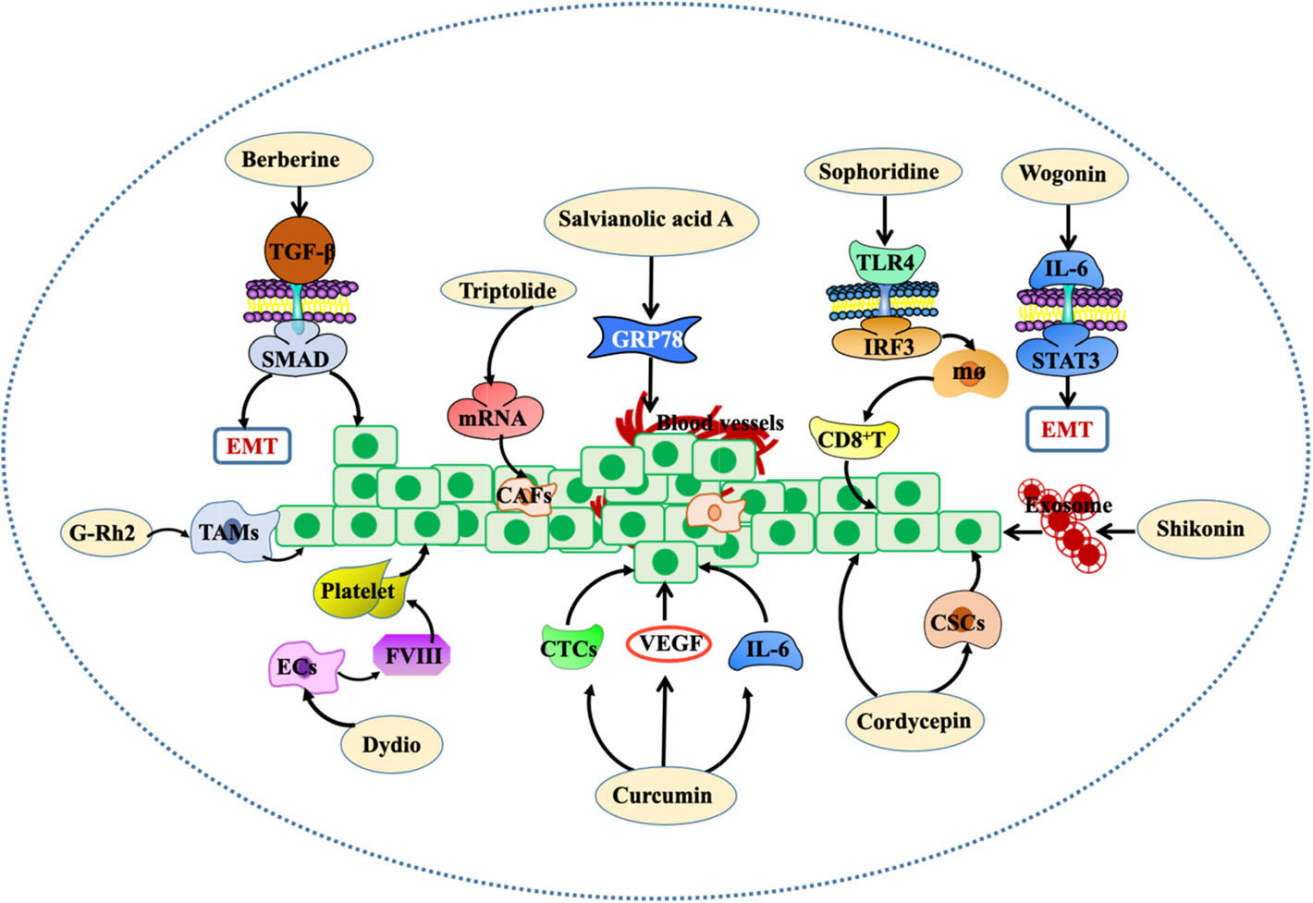
Intervention of natural products from traditional Chinese medicine on the TME. Curcumin inhibits VEGF, IL-6 and cancer stem cells in vivo and in vitro. Ginsenoside Rh2 (G-Rh2) can regulate the phenotype of TAMs to improve the TME. Dihydrodiosgenin (DYDIO) can inhibit platelet activation and reduce endothelial cell-derived factor VIII (FVIII). Cordycepin can target CSCs and upregulate the apoptosis of cancer cells. Shikonin reduces cancer-derived exosomes to inhibit the spread of breast cancer cell lines. Wogonin inhibits the transformation of EMT into the epithelial stroma by interfering with the IL-6/STAT3 signalling pathway. Sophoridine can inhibit macrophage-mediated immunosuppression through the TLR4/IRF3 pathway and then upregulate the killing effect of CD8^+^ T cells on gastric cancer. Salvianolic acid A can block the secretion of glucose-regulated protein 78 (GRP78) and inhibit angiogenesis. Triptolide (TP) inhibits the proliferation of cancer cells. Berberine can mediate the transforming growth factor-β signalling pathway, thus inhibiting EMT and promoting apoptosis

Traditional Chinese medicine (TCM) has a long history and unique advantages. Based on the needs of the comprehensive treatment of clinical cancers, various natural products from TCM have been investigated in the therapy of cancers (Fig. 6).

Curcumin (diferuloylmethane) is an active ingredient in plant turmeric spices [152]. It suppresses tumour progression by affecting many aspects of the microenvironment. Based on in vivo and in vitro studies of ovarian cancer, curcumin interferes with the TME and affects tumour progression by inhibiting transcription factor nuclear factor-NB (NF-NB), signal transduction, activation of transcriptional activator 3 and expression of angiogenic cytokines [110]. For pancreatic cancer, preclinical studies have found that a new curcumin synthesis derivative (CDF) can inhibit VEGF, IL-6 and tumour stem cells, thus affecting the TME of pancreatic cancer [111]. In addition, a randomized phase 1 placebo-controlled trial of the APG-157 botanical drug made from curcumin was conducted. A total of 13 normal subjects and 12 oral cancer subjects participated in the study, of which 12 were treated with placebo and 13 were treated with the active drug APG-157. It was found that the infiltrating expression of immune cells (CD4^+^ and CD8^+^ cells) and the expression of PD-1 and PD-L1 were significantly increased in patients receiving APG-157, implying that APG-157 therapy has the potential to attract immune cells into the TME and provides a strong theoretical basis for using immune checkpoints to block the interaction between T cells and cancer cells (PD-1/PD-L1 axis) [112]. In a randomized, double-blind, placebo-controlled trial, 97 patients were randomly divided into a curcumin group (*n* = 49) and a placebo group (*n* = 48). Oral curcumin for 6 months had no significant effect on the overall withdrawal time of prostate cancer patients with intermittent androgen deprivation, and curcumin intake inhibited the increase in prostate-specific antigen (PSA) during curcumin administration. It has been suggested that curcumin has a potential beneficial effect on patients with prostate cancer [113]. Curcumin not only intervenes the TME but also improves the safety and tolerance of patients to a certain extent, suggesting that while traditional Chinese medicine plays an anti-cancer role, it is also helpful for the self-protection of patients.

By interfering with the target or signalling pathway in the microenvironment, TCM can inhibit the microenvironment aiding cancer through immunosuppression, the EMT and cancer stem cells. Through in vitro experiments, it was found that sophoridine can inhibit macrophage-mediated immunosuppression through the TLR4/IRF3 pathway and then up-regulate the killing effect of CD8^+^ T cells on gastric cancer, thus reshaping the immune microenvironment of gastric cancer, which provides a preclinical basis for the clinical application of sophoridine [114]. Research by Li et al. indicated that ginsenoside Rh2 (G-Rh2) can improve the TME by regulating the phenotype of TAMs in lung cancer tissues [115]. Huang et al. demonstrated that berberine can mediate Smad-independent and Smad-dependent TGF-β signalling pathways, thereby inhibiting EMT and promoting apoptosis [116]. Zhao et al. found that wogonin inhibits EMT in the inflammatory microenvironment by interfering with the IL-6/STAT3 signalling pathway in mice with lung cancer [153]. Li et al. found that for the mouse model of metastasis of human colon cancer, Bigelovin inhibits the EMT by interfering with the expression of N-and E-cadherin, inhibits colony formation through the STAT3 pathway, and reduces cell invasion through the cofilin pathway [117]. Jin et al. demonstrated that cordycepin regulates the TME and inhibits cancer growth by targeting CSCs, upregulating cancer cell apoptosis and eliciting cell cycle arrest [118].

The TCM can reduce or block the communication between cells in the TME, thus destroying the microenvironment and inhibiting cancer metastasis. The TCM can affect the communication between cells in the TME, thus inhibiting cancer proliferation. Wei et al. provided evidence that shikonin checks the diffusion of a breast cancer cell line (MCF-7) by reducing the exosome of cancer origin [119]. Through in vivo experiments, Liu et al. showed that the triptolide exosome delivery system (TP-Exos) can reduce the apoptosis and cytotoxicity of TP on SKOV3 cells and enhance the inhibitory effect of TP on cell proliferation [154].

Some studies have also shown the effect of natural products from TCM on improving the TME by promoting vascular normalization. Zhong et al. found that 6-gingerol (6G) reduces cancer growth and metastasis by promoting cancer vascular normalization, reducing microvascular structure entropy (MSE), improving the TME and reducing cancer metastasis in a patient-derived cancer xenotransplantation (PDTX) model [155]. Yang et al. demonstrated that salvianolic acid A can block the secretion of glucose-regulated protein 78 (GRP78) to inhibit cancer-related angiogenesis [156]. Zhuang et al. demonstrated that dihydrodiosgenin (DYDIO) can inhibit platelet activation and reduce endothelial cell-derived factor VIII (FVIII) to inhibit HCC metastasis [157].

There are many types of TCMs that can act on different signalling pathways in the TME, inhibit cancer recurrence and metastasis, have high safety, and can be treated according to the patient’s physique, syndrome differentiation and precise treatment. However, there are few modern pharmacological studies of TCM, and most of them are preclinical studies. Therefore, more in-depth exploration is needed for broad clinical practice.

### Combination therapy for the TME

**Fig. 7:**
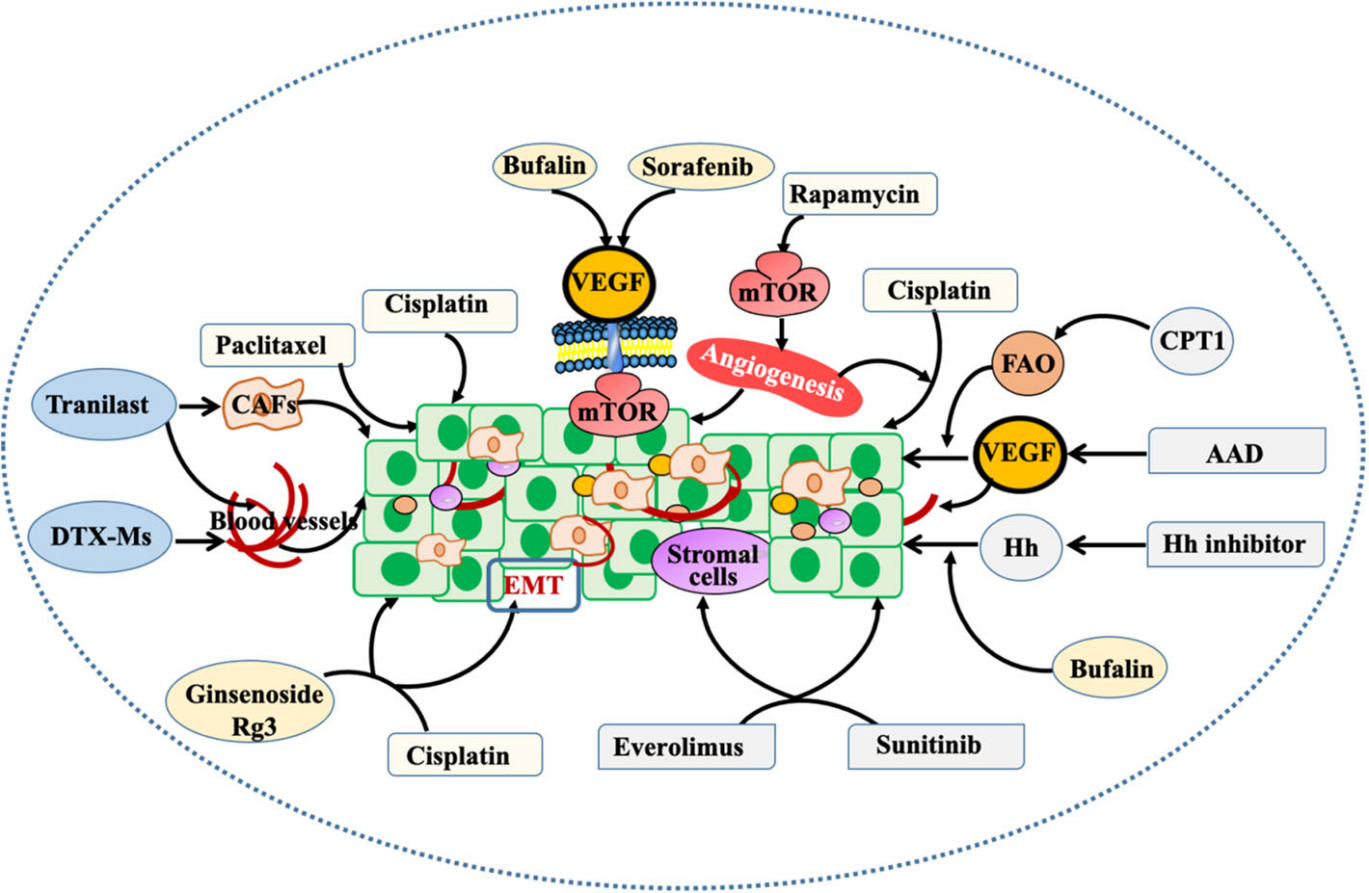
Intervention of combined drugs on the TME. Rapamycin (RapA) is a mTOR inhibitor that inhibits tumour proliferation through anti-angiogenesis and can be enhanced in combination with cisplatin. Cisplatin combined with paclitaxel inhibits tumour invasion. The combination of everolimus and sunitinib can affect stromal cells and cancer cells in the TME. Antiangiogenic drugs (AADs) combined with carnitine palmitoyltransferase 1A (CPT1) inhibitors significantly inhibited fatty acid oxidation (FAO) -induced cell proliferation and angiogenesis. Ginsenoside Rg3 combined with cisplatin can inhibit epithelial-mesenchymal transition (EMT) of tumour cells. Hedgehog (HH) signalling pathway inhibitors combined with bufalin can inhibit tumour proliferation. Tranilast can dow-regulate CAF activity, promote vascular normalization, and help docetaxel micelles (DTX-ms) reach tumour tissue through veins and kill tumour cells. Sorafenib combined with bufalin affects the tumour vascular microenvironment through targeting the mTOR/VEGF signalling pathway

To better intervene in the TME, combination therapy may be a good strategy, such as the combination of two different chemotherapy drugs or a combination of chemotherapy drugs and natural products from TCM (Fig. 7).

#### Combination of chemotherapy

Chemotherapeutic drugs used in combination with other types of drugs or different chemotherapeutic drugs have a good effect on improving the TME. For example, Guo et al. found that rapamycin (RapA) is a mTOR inhibitor that can provide significant efficacy in the treatment of melanoma in xenograft models through antiangiogenic activity and can be co-cultured with cisplatin because RAPA makes A375 melanoma cells sensitive to cisplatin through microenvironmental regulation [122]. Chemotherapy resistance is widely considered to be one of the main factors that limit the therapeutic effect of cancer patients and affect the clinical outcome. In the first phase clinical trial, Heeren et al. proved that cisplatin combined with paclitaxel has a stronger regulatory effect on cancer invasion than cisplatin alone [158].

#### Combination of targeted therapy

The combination of targeted drugs can interfere with the TME and inhibit cancer growth and invasion from many aspects and multiple targets. The results of Kitano et al. suggested that the combination of everolimus and sunitinib effectively inhibited the TME composed of mesenchymal and cancer cells in renal carcinoma models [159].

Targeting cancer angiogenesis is a good intervention method. Interestingly, in the fatty liver model of liver metastasis of liver cancer and colon cancer, Iwamoto et al. discovered that cancer hypoxia induced by antiangiogenic drugs (AADs) can initiate fatty acid oxidative metabolism reprogramming, increase free fatty acid (FFA) uptake, and thus stimulate cancer cell proliferation. Reducing carnitine palmitoyltransferase 1A (CPT1) can significantly inhibit cell proliferation induced by FFA. Therefore, the deletion of CPT1 can enhance the therapeutic effect of AAD and its anti-cancer effect [160].

In addition, the combination of poly (adenosine diphosphate-ribose) polymerase (PARP) inhibitor and mitogen-activated protein kinase (MEK) inhibitor produces a synergistic cytotoxic effect in a variety of RAS mutant tumor models in vitro and in vivo. It induces BIM-mediated apoptosis by activating caspase-3, inhibits the expression of CD31 in endothelial cells, and inhibits the production of mutant RAS-induced VEGF through the RAS/MAPK pathway, thus affecting the vascular microenvironment [120]. PARP inhibitors were the first cancer drug to synthesize lethal targeted therapy and the first clinically approved drug to take advantage of synthetic lethal advantages [121]. Synthetic lethality (SL) is a concept put forward by geneticists nearly a century ago to describe a situation in which either defect in two genes has little effect on a cell or organism, but a combination of defects in the two genes may lead to death [161]. For example, a prospective phase III trial (POLO trial, ongoing pancreatic cancer Oraparib, NCT02184195) was recently conducted to evaluate the efficacy of olapril in patients with BRCA mutation and metastatic PDAC [162]. Furthermore, preclinical trials showed that PARPI could upregulate PD-L1 expression directly or indirectly by promoting the activation of the IFN pathway [123, 124].

#### Combined with TCM therapy

In an open-label randomized controlled trial, Lynne et al. found that curcumin combined with the FOLFOX regimen in the treatment of stage IIa metastatic colorectal cancer can improve the safety and tolerance of cancer patients and improve the therapeutic effect [163]. In addition, Sheng et al. also proposed their own point of view, indicating that the combination of hedgehog (Hedgehog, HH) signal pathway inhibitors and bufalin can significantly reduce the malignant biological behaviour of liver cancer [164]. Wang et al. found that sorafenib combined with bufalin affected the cancer vascular microenvironment by targeting the mTOR/VEGF signalling pathway in mice with liver cancer, thus exerting a synergistic anti-liver cancer effect [165]. In solid cancers, hypoxia changes the microenvironment and is related to proliferation, metastasis and drug sensitivity. Wang et al. showed that ginsenoside Rg3 combined with cisplatin can significantly reverse the dryness and EMT of non-small-cell lung cancer (NSCLC) cells induced by hypoxia in vivo and in vitro [166].

#### Others

Additionally, the combination of other cancer therapies also has a good effect on the TME. Pang et al. discovered that pre-administration of tranilast can downregulate the activity of CAFs, cut off the relationship between cancer cells and CAFs, and facilitate vascular normalization. Docetaxel micelles (DTX-ms) then pass through nearby veins to reach the cancer tissue. Due to the improved retention (EPR) effect and permeability, the micelles were passively trapped in the cancer and further spread to the interior to kill cancer cells [167].

By exerting the synergistic therapeutic effect of drugs, combined drugs can interfere with the TME from many aspects (such as immunity, angiogenesis, EMT or hypoxia), destroy the soil of cancer growth and metastasis to improve treatment efficacy, inhibit drug resistance and prevent cancer metastasis. This is indispensable in the mode of the comprehensive treatment of cancers.

## Conclusions and perspectives

TME is the soil of cancer growth and metastasis. The primary TME promotes cancer growth and development, and the pre-metastatic microenvironment provides the end point for disseminated cancer cells and prepares for cancer metastasis, while the metastatic microenvironment awakens dormant disseminated cancer cells and finally forms metastatic foci. Therefore, when taking the TME as the intervention target, it is best to detect the specific state of the current TME, such as detecting immune markers, and introduce therapeutic drugs at the right time. Only in this way can we better inhibit or even block the invasion and metastasis of the cancer and provide the basis for the further elimination of the cancer. Of course, the detection of the TME is still a problem that may be solved by measuring cells, body fluids and cytokines in serum.

Currently, the intervention methods for the TME include chemotherapy, targeted therapy, immunotherapy, TCM therapy and so on. Compared with single therapy, combined therapy can interfere with multiple targets in the TME at the same time to achieve the effect of cooperative therapy, quickly destroy the interaction between the TME and cancer cells, and prevent and treat cancer metastasis. However, these have yet to be verified in clinical trials. Moreover, it is not clear how to use drugs in combination, such as the conditions of combined use, the antagonism between drugs, the order before and after combined use, and so on. Although these studies still have a variety of limitations, with the arrival of the era of the comprehensive treatment of the cancer model, combined therapy intervention of the TME has a good development prospects.

## Data Availability

Not applicable.

## Abbreviations

TME: Tumor microenvironment
VECs: Vascular endothelial cells
VEGF: Vascular endothelial growth factor
ECM: Extracellular matrix
CAFs: Cancer-associated fibroblasts
TAMs: Tumor-associated macrophages
BMDCs: Bone marrow-derived dendritic cells
MDSCs: Myeloid-derived suppressor cells
FGF: Fibroblast growth factor
EGF: Epidermal growth factor
PDGF: Platelet-derived growth factor
TGF: Transforming growth factor
HSC: Hepatic stellate cells
ANG-1: Angiopoietin-1
EVs: Extracellular vesicles
ITGBL1: Integrin β1
CRC: Colorectal cancer
HIC-5: Hydrogen peroxide-induced clone 5
ESCC: Esophageal squamous cell carcinoma
EMT: Epithelial-mesenchymal transition
MSCs: Mesenchymal stem cell
GALNT14: N-acetylgalactosamine transferase 14
PMN: Pre-metastatic niche
eATP: extracellular ATP
DTCs: Disseminated tumor cells
TF: Tissue factor
LOX: Lysyl oxidase
HPC: Hematopoietic progenitor cell
OXP: Oxaliplatin
PDAC: Pancreatic ductal adenocarcinoma
XIST: X-inactivated specific transcript
mCRPC: metastatic castration resistance to prostate cancer
PFS: Progression-free survival
BTZ: Bortezomib
PST: Penobarbital
PSS: Propylene glycol alginate sodium sulfate
DOX: Doxorubicin
HCC: Hepatocellular carcinoma
RPS15A: Ribosomal protein s15a
CTLA-4: Cytotoxic T lymphocyte associated protein 4
PD-1: Programmed cell death protein 1
CTCs: Circulating tumor cells
TCM: Traditional Chinese medicine
DYDIO: Dihydrodiosgenin
APS: Astragalus polysaccharide
ER: Endoplasmic reticulum
MSE: Microvascular structure entropy
RAPA: Rapamycin
AAD: Antiangiogenic drug
CPT1: Carnitine palmitoyltransferase 1A
NSCLC: Non-small cell lung cancer
